# The pedunculopontine tegmentum controls renal sympathetic nerve activity and cardiorespiratory activities in nembutal-anesthetized rats

**DOI:** 10.1371/journal.pone.0187956

**Published:** 2017-11-09

**Authors:** Anne M. Fink, Caron Dean, Mariann R. Piano, David W. Carley

**Affiliations:** 1 Center for Narcolepsy, Sleep, and Health Research, University of Illinois at Chicago, Chicago, Illinois, United States of America; 2 Department of Biobehavioral Health Science, University of Illinois at Chicago, Chicago, Illinois, United States of America; 3 Department of Anesthesiology, Medical College of Wisconsin and Zablocki VA Medical Center, Milwaukee, Wisconsin, United States of America; 4 Department of Bioengineering, University of Illinois at Chicago, Chicago, Illinois, United States of America; Max Delbruck Centrum fur Molekulare Medizin Berlin Buch, GERMANY

## Abstract

Elevated renal sympathetic nerve activity (RSNA) accompanies a variety of complex disorders, including obstructive sleep apnea, heart failure, and chronic kidney disease. Understanding pathophysiologic renal mechanisms is important for determining why hypertension is both a common sequelae and a predisposing factor of these disorders. The role of the brainstem in regulating RSNA remains incompletely understood. The pedunculopontine tegmentum (PPT) is known for regulating behaviors including alertness, locomotion, and rapid eye movement sleep. Activation of PPT neurons in anesthetized rats was previously found to increase splanchnic sympathetic nerve activity and blood pressure, in addition to altering breathing. The present study is the first investigation of the PPT and its potential role in regulating RSNA. Microinjections of DL-homocysteic acid (DLH) were used to probe the PPT in 100-μm increments in Nembutal-anesthetized rats to identify effective sites, defined as locations where changes in RSNA could be evoked. A total of 239 DLH microinjections were made in 18 rats, which identified 20 effective sites (each confirmed by the ability to evoke a repeatable sympathoexcitatory response). Peak increases in RSNA occurred within 10–20 seconds of PPT activation, with RSNA increasing by 104.5 ± 68.4% (mean ± standard deviation) from baseline. Mean arterial pressure remained significantly elevated for 30 seconds, increasing from 101.6 ± 18.6 mmHg to 135.9 ± 36.4 mmHg. DLH microinjections also increased respiratory rate and minute ventilation. The effective sites were found throughout the rostal-caudal extent of the PPT with most located in the dorsal regions of the nucleus. The majority of PPT locations tested with DLH microinjections did not alter RSNA (179 sites), suggesting that the neurons that confer renal sympathoexcitatory functions comprise a small component of the PPT. The study also underscores the importance of further investigation to determine whether sympathoexcitatory PPT neurons contribute to adverse renal and cardiovascular consequences of diseases such as obstructive sleep apnea and heart failure.

## Introduction

Elevated renal sympathetic nerve activity (RSNA) accompanies a variety of disorders, including obstructive sleep apnea, heart failure, and chronic kidney disease [1–3]. Renal sympathetic efferent and afferent nerves transmit signals leading to release of catecholamines, changes in renal blood flow, and alterations in sodium/fluid homeostasis. Chronically elevated RSNA contributes to hypertension. Blood pressure (BP) can be reduced by targeting pathophysiologic renal mechanisms, including RSNA [4]. For example, ablation of renal sympathetic nerve fibers reduced BP in individuals who did not respond to pharmacologic therapies [5]. This approach is controversial, however, because the underlying cause of sympathetic over-activity remains untreated. Considering that a constellation of different disorders involve elevated RSNA, it is important to investigate the brainstem mechanisms linking these conditions.

Hypertension and renal dysfunction are prevalent comorbidities with obstructive sleep apnea [6–8]. This observation suggests that the brain regions known to control sleep and breathing could also directly control renal function and contribute to renal/vascular pathologies, providing new targets for therapies treating cardiovascular diseases, particularly those evoked or exacerbated by sleep-disordered breathing. The present study was conducted to investigate the pedunculopontine tegmentum (PPT), a cluster of heterogeneous neurons at the midbrain-pontine junction that are a source of ascending and descending cholinergic pathways to cortical and brainstem structures (e.g., thalamus, locus coeruleus, pontine reticular formation, basal ganglia) [9,10]. The PPT innervates and receives inputs from structures that control arousal/alertness, rapid eye movement (REM) sleep, breathing, and movement/locomotion [11–16]. The anatomical position of the PPT supports a role in autonomic regulation, with a descending cholinergic projection from the PPT to the rostral ventrolateral medulla (RVLM) that contains pre-ganglionic sympathetic neurons [17,18]. In addition, the PPT receives baroreceptor and chemoreceptor signals from the solitary nucleus, indicating a role in the modulation of cardiorespiratory reflexes [19]. Retrograde neuronal tracing studies suggest a possible paucisynaptic connection between the PPT and the kidneys [20,21], but there have been no studies measuring RSNA in response to PPT stimulation. The purpose of the present study was to determine how microinjecting DL-homocysteic acid (DLH) into the PPT alters RSNA. We tested the hypothesis that PPT activation can elevate RSNA in spontaneously breathing Nembutal-anesthetized rats.

## Materials and methods

### Surgical procedures

**Fig 1:**
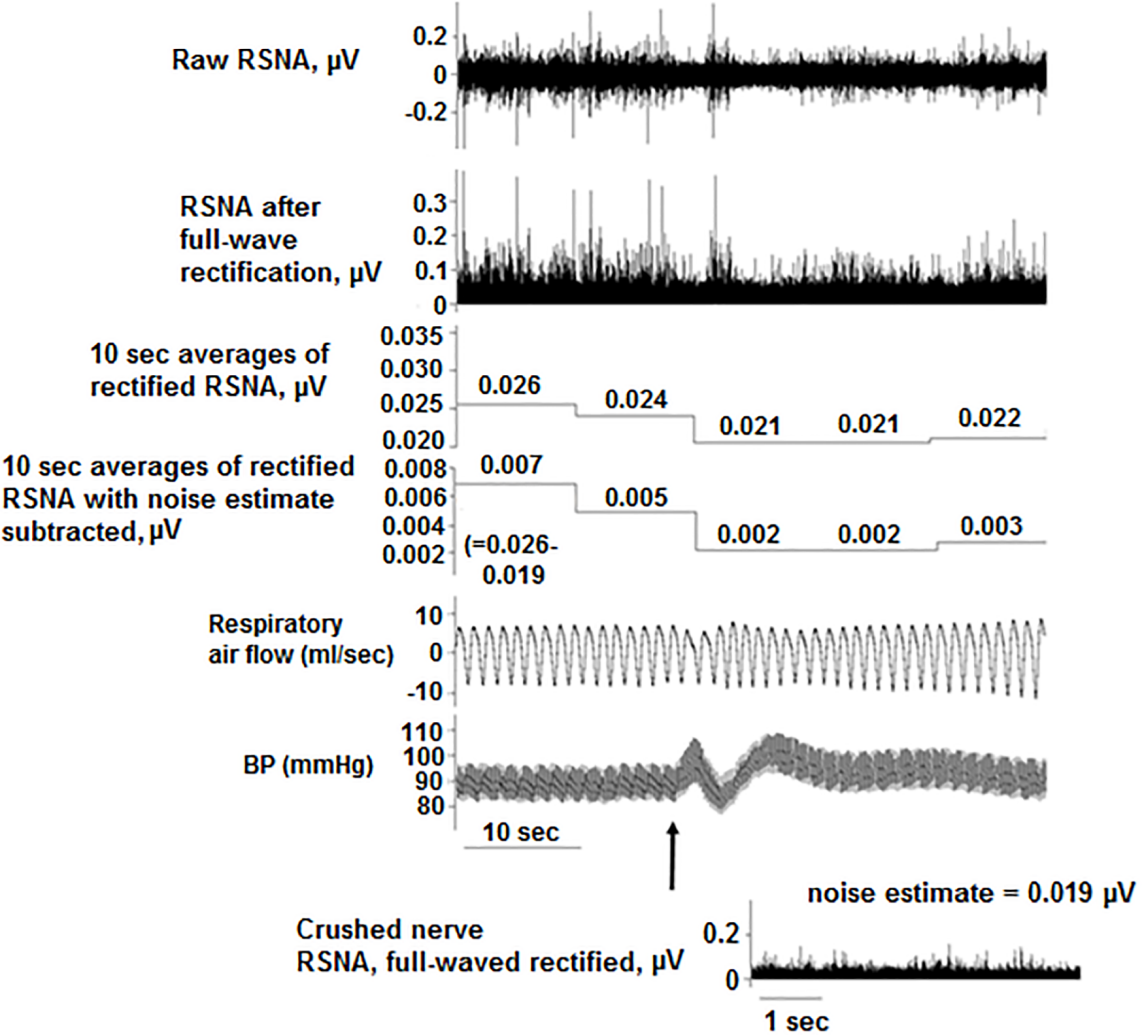
RSNA response to intravenous saline. After undergoing full-wave rectification, the RSNA was averaged in non-overlapping 10-second intervals. The average noise (determined by crushing the nerve bundle at the end of the experiment) was subtracted from each 10-second segment. The arrow denotes an IV bolus of saline (1 ml, infused over 3 sec) and the resulting physiologic responses.

**Fig 2:**
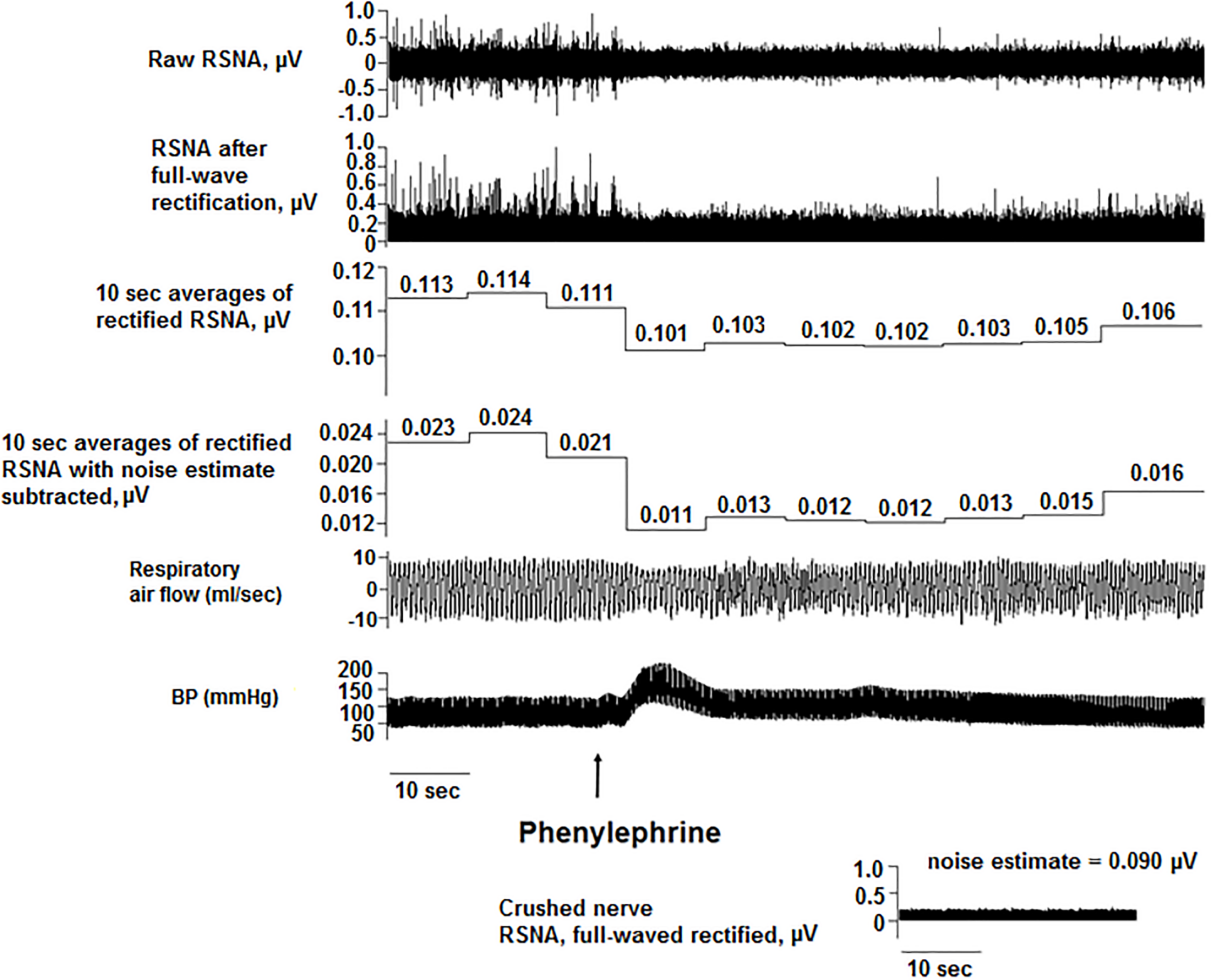
Renal sympathoinhibition in response to phenylephrine injection. Phenylephrine (1 μg/ml) was injected intravenously to assess RSNA inhibition. The raw RSNA was full-wave rectified, and non-overlapping 10-second averages (minus noise) were calculated to demonstrate inhibition in RSNA. The arrow denotes the timing of the phenylephrine injection.

**Fig 3:**
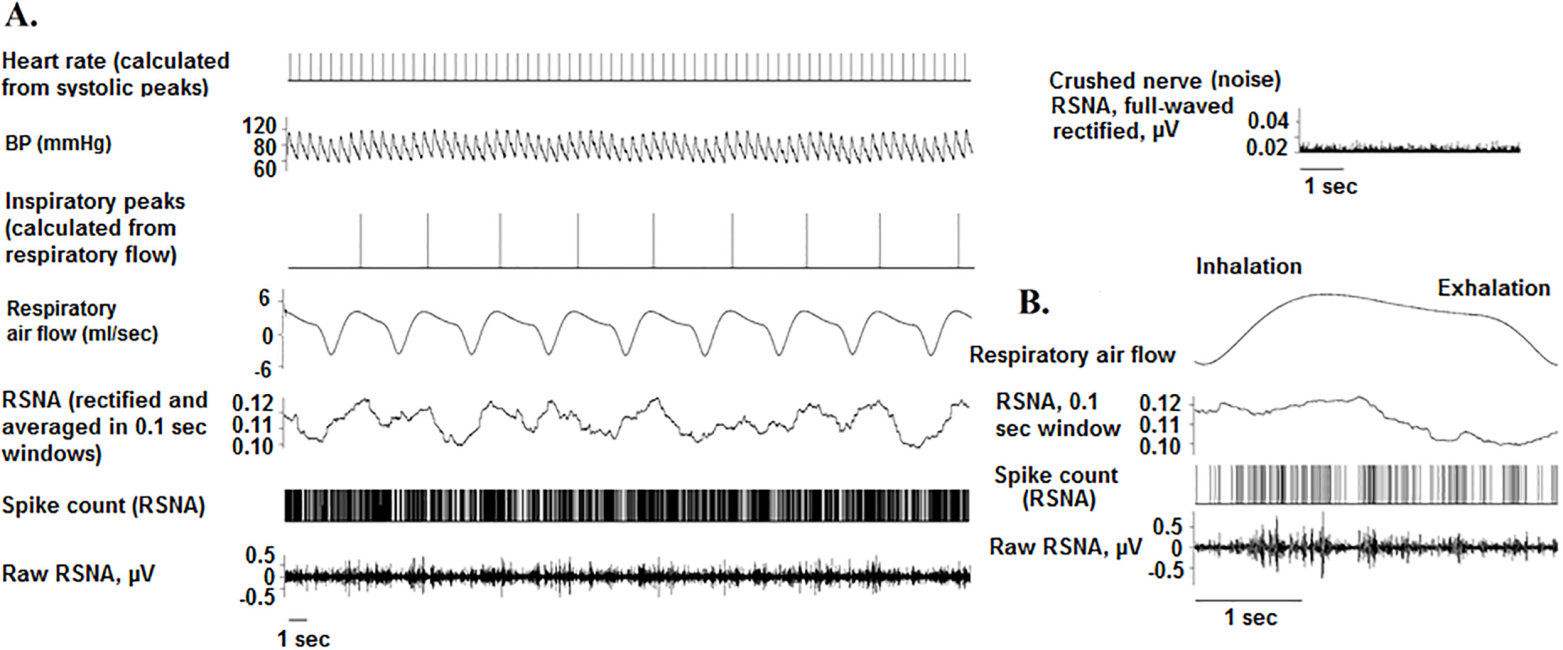
Respiratory modulation of RSNA. Panel A illustrates a representative example of BP, respiratory flow, and RSNA data from a rat. The full-wave rectified RSNA was averaged in a 0.1 second window. RSNA activation is evident during inspiration. Panel B illustrates the characteristic RSNA pattern for a single breath.

Experiments were performed in adult, male Sprague-Dawley rats (*N* = 18, 280–330 g; Harlan/Envigo Laboratories, Indianapolis, IN). Procedures conformed to the American Physiological Society’s *Guiding Principles for the Care and Use of Vertebrate Animals* [22] and were approved by the University of Illinois at Chicago Institutional Animal Care and Use Committee. Similar to other studies [16,23–25], rats were anesthetized with Nembutal (pentobarbital sodium, 50 mg/kg intraperitoneally). In our previous studies, this anesthetic achieved a sustained plane of anesthesia, and it did not interfere with the modulation of RSNA for the duration of the experiment [26–28]. Body temperature was maintained at 37°C with a heating pad. A catheter was placed in the femoral vein, and, when necessary, supplemental Nembutal doses were given (2 mg/kg intravenously). BP was measured in the femoral artery (telemetry transmitter model PA-C10, Data Sciences International, Minneapolis, MN). The trachea was cannulated with polyethylene tubing (Braintree Scientific Inc., Braintree, MA) and connected to a pneumotachograph (BIOPAC Systems Inc., Model TSD237B, Goleta, CA) to measure respiratory air flow while breathing room air spontaneously [29]. A renal sympathetic nerve was exposed retroperitoneally, placed on bipolar stainless steel wire electrodes (A-M Systems, Carlsborg, WA), and fixed in position with silicone gel (Wacker, München, Germany) as previously described [26,28,30]. The RSNA signals were directed to a 10x pre-amplifier and microelectrode amplifier (Model 1800, A-M Systems, Carlsborg, WA) using high- and low-pass filtering (10 Hz and 3 kHz), gain up to 10 K (4000 Hz sampling rate). All physiologic data were acquired and A/D converted using DataWave/SciWorks data acquisition software (Loveland, CO), which displayed a raw and a full-wave rectified RSNA. After recordings, signals were analyzed using Spike 2 (Cambridge Electronic Design, Cambridge, UK), given the range of analysis tools provided by this software for RSNA. Before probing the PPT with microinjections of DLH, the quality of each RSNA recording was assessed by evoking the baroreflex; rats were given intravenous injections of saline (1 ml bolus; Fig 1) or phenylephrine (10 μg/ml; Fig 2) to increase BP and produce sympathoinhibition and bradycardia. Recordings were also assessed for evidence of respiratory modulation of the RSNA, indicated by RSNA activation during early inspiration [31] (Fig 3). At the end of experiments, the noise level in RSNA was determined by crushing the nerve proximal to the recording electrodes.

### DLH microinjections

Glass micropipettes/electrodes were built by fusing 1 mm x 0.25 mm glass barrels while pulling the glass to an overall tip diameter of 10–20 μm (A-M systems, Carlsborg, WA), which allowed microinjection of multiple agents at a single site in the brain [15]. Micropipette barrels were filled with DLH (4 mmol/L, dissolved in phosphate-buffered saline [PBS], Sigma-Aldrich Co., St. Louis, MO), a control/vehicle solution (PBS, 0.1 mol/L, pH = 7.4), Oil Red-O dye (7 mg in 1 ml ethanol, Sigma-Aldrich Co., St. Louis, MO), and pontamine sky blue dye (1% in PBS, Sigma-Aldrich Co., St. Louis, MO).

**Fig 4:**
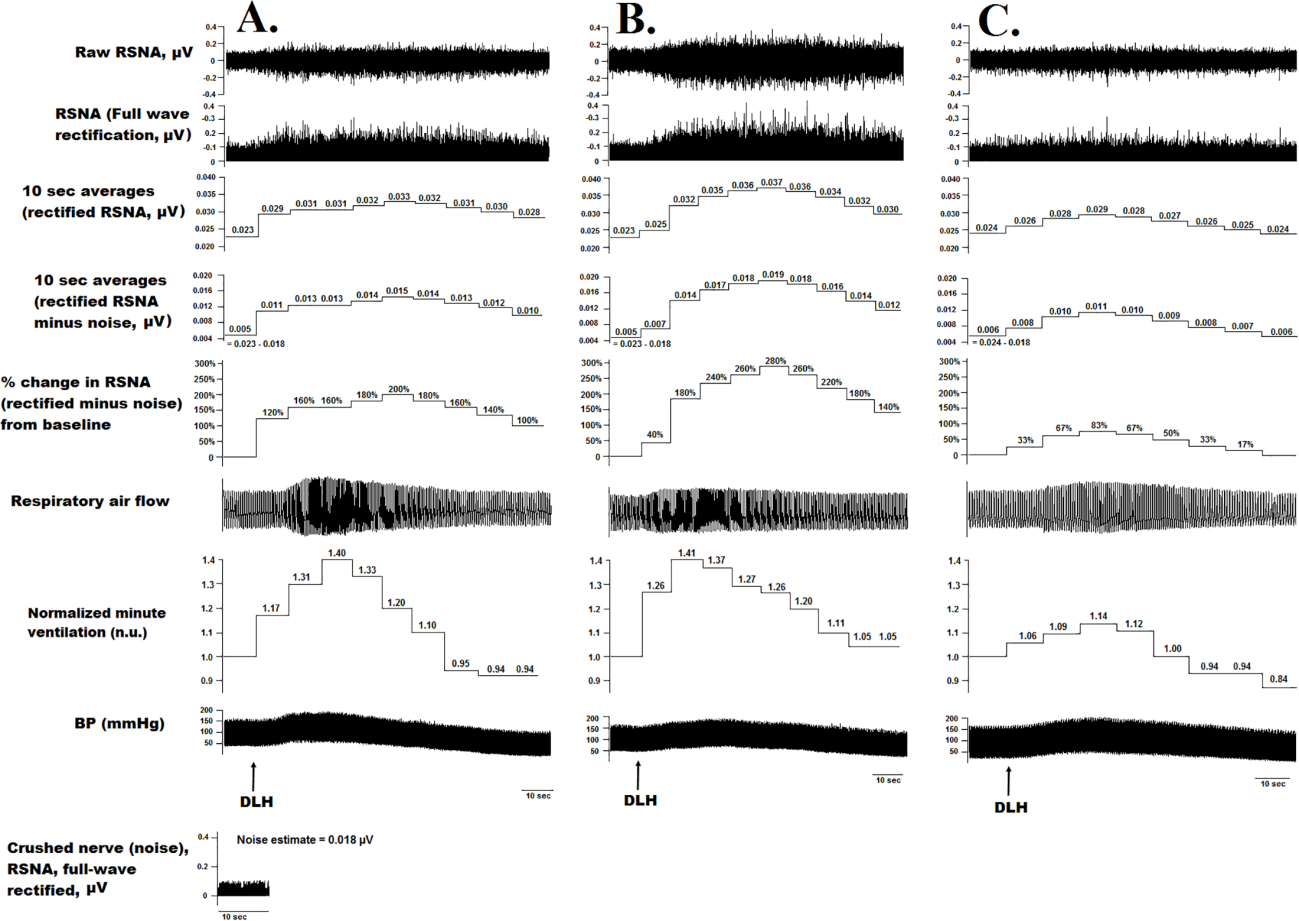
Repeated DLH microinjections into the same PPT location evoke renal sympathoexcitation, increased BP, and ventilatory changes. Panels A, B, and C represent responses with 5 minutes separating activation of the same PPT location. The three arrows denote the timing of 10 nL DLH pressure microinjections into the PPT.

Rats were placed in a stereotaxic frame (Stoelting Co., Wood Dale, IL), and the bregma was used as a landmark for targeting the coordinates of the PPT (6.8–8.6 mm caudal and 1.4–2.2 mm lateral from the bregma) [32]. A computer-controlled manipulator (StereoDrive, Neurostar, Tubingen, Germany) was used to position the tip of the glass micropipette at the dorsal margin of the PPT (6.5 mm below the surface of the brain) via a burr-hole osteotomy. The PPT was probed with microinjections of DLH (10 nL over 100 ms under ~70 psi) using a 100-μm distance between injections. Effective sites were defined as locations where changes in RSNA could be evoked and reproduced by at least two additional DLH microinjections (Fig 4) after baseline parameters were regained. After ensuring reproducibility, control injections of PBS (10 nL) ensured that no responses were elicited by this vehicle solution. The PPT was probed bilaterally on a 100-μm grid basis, with experiments terminated if rats demonstrated evidence of hemodynamic instability (i.e., a ≥ 20% decrease in mean arterial pressure or respiratory rate) or if body temperature was no longer maintained at 37°C. The microinjector (MMPI-3, ASI Applied Scientific Instrumentation, Eugene, OR) provided a logic level pulse in the recordings (used as a zero marker for analyses) to indicate the timing of DLH microinjections; there were 3–5 minute intervals between consecutive microinjections, and volumes were quantified by measuring the change in meniscus level using a microscope with an eyepiece reticule.

### Data and statistical analysis

**Fig 5:**
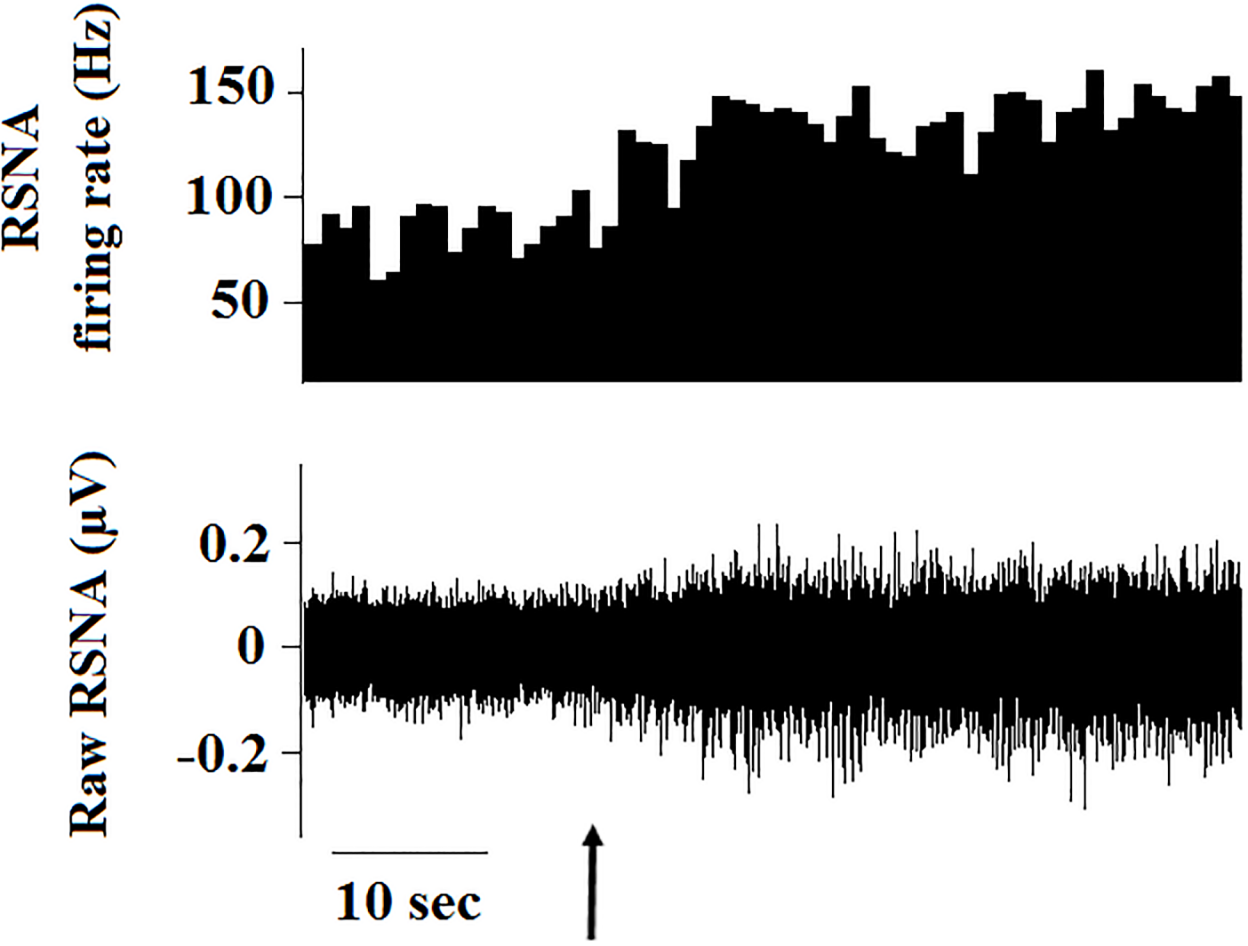
Rate-meter analysis illustrating increased firing rate (spikes per second). Renal sympathoexcitation was evoked by microinjecting DLH (arrow) into the PPT.

For each effective site, average values for RSNA, mean arterial pressure, heart rate, respiratory rate, normalized tidal volume, and normalized minute ventilation were calculated for the 10-second baseline preceding the DLH microinjection and for 10-second segments up to 1 minute after PPT activation. Although effective sites underwent additional testing to ensure their reproducibility, only the initial evoked response was included in the collective statistical analyses to avoid the potential confounding influence of re-activating the same neurons in the same animal. Two methods were employed for analyzing RSNA in each 10-second segment; one focused on measuring changes in voltage, and the other focused on assessing firing rate. Absolute values were obtained by full-wave rectifying the raw RSNA signal. Averages for each non-overlapping contiguous 10-second segment were calculated. The noise (full-wave rectified values averaged for a 10-second segment of the “crushed” nerve) for each rat was subtracted from the full-wave rectified RSNA averages. Values were expressed as a percent change from baseline (with baseline value set to 0%) to control for variations in basal sympathetic tone among animals [31]. In addition to this approach, the frequency of RSNA firing was measured using a custom Spike 2 script (Cambridge Electronic Design, Cambridge, UK) that applied an amplitude discriminator to detect the number of peaks in the raw signal, as illustrated in the example in Fig 5, where the height of each vertical bar is proportional to the spikes per second.

The BP signal was used to determine mean arterial pressure and heart rate, which were calculated by the DataWave/SciWorks software for each interval. The respiratory flow signal was analyzed to detect the number of breaths in each 10-second interval, which was multiplied by 6 to reflect respiratory rate/minute. Changes in tidal volume were calculated by measuring the area under the curve (AUC) for each breath and aggregating these data into averages for each 10-second segment. Using a normalization approach reported previously [33], the average for each 10-second interval was divided by the baseline value (setting baseline to 1 n.u. [normalized units]) to reflect changes in the depth of breathing. Normalized minute ventilation values were calculated by multiplying normalized tidal volume by the respiratory rate (setting baseline to 1 n.u.). Repeated-measures analysis of variance with Šidák correction for multiple comparisons (SPSS, Version 24, IBM, Chicago, IL) was used to determine which 10-second periods differed with respect to baseline for each variable. Data are reported as mean ± standard deviation; *p* ≤ 0.05 was set for statistical significance.

### Histology and verification of effective site locations

Effective sites were marked with Oil Red-O or pontamine sky blue dye; the use of two dyes permitted the differentiation of multiple effective sites in the event that they were within close proximity. At the end of the experiment, rats underwent intracardiac perfusion with 0.9% saline followed by 4% paraformaldehyde. The brain was removed, post-fixed, and frozen. Serial frozen 20-μm sections were cut in the coronal plane for histological localization of microinjection sites. Sections were incubated in nicotinamide adenine dinucleotide phosphate (NADPH) and nitroblue tetrazolium (Sigma-Aldrich Co., St. Louis, MO) to label NADPH-diaphoresis-positive cells [34], a marker conventionally used to identify the PPT [35].

## Results

**Fig 6:**
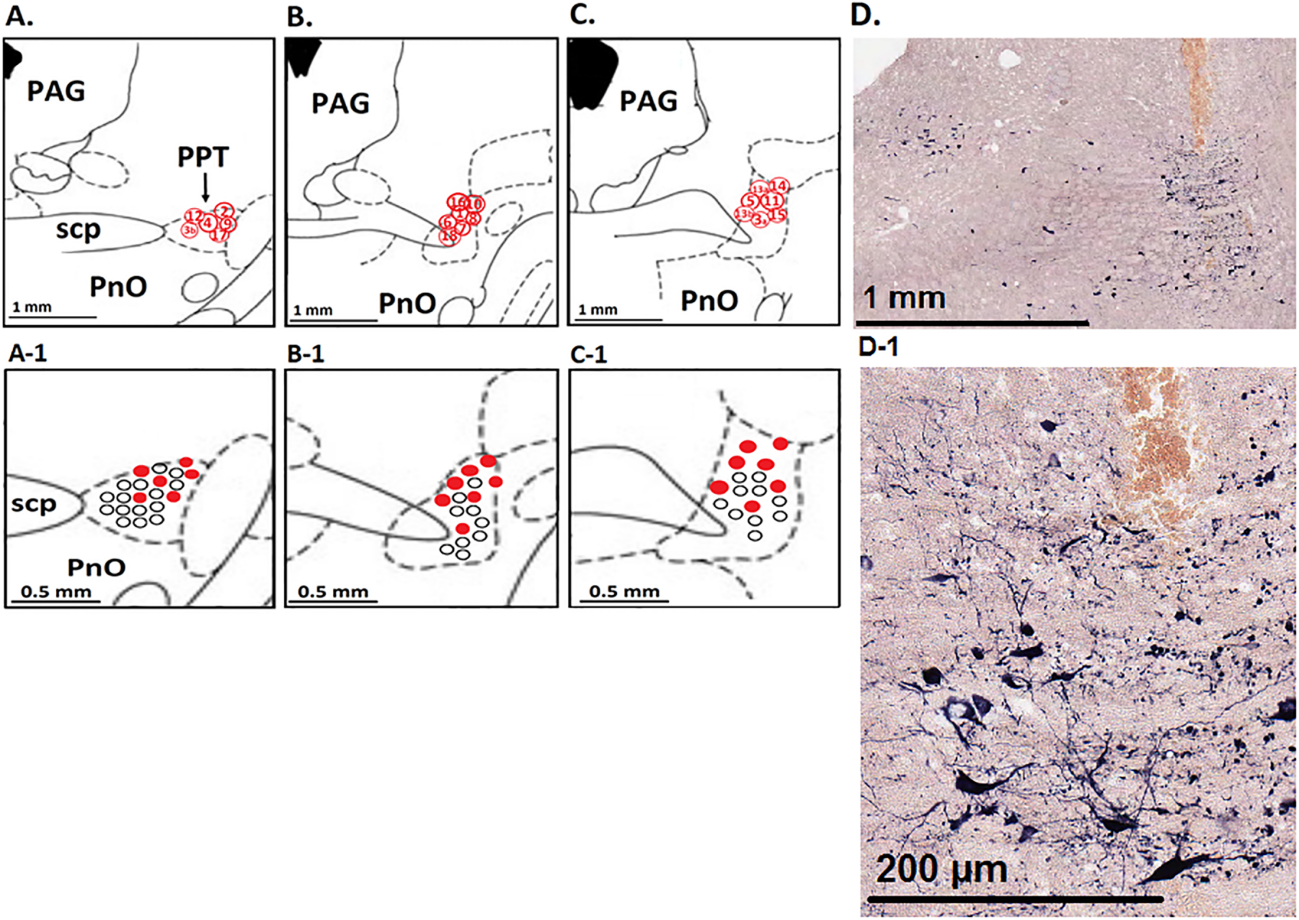
Diagrammatic representation of effective and ineffective sites in the PPT. This semischematic drawing shows three coronal sections of the pontine reticular nuclei. Panels A, B, and C are based on diagrams from Paxinos and Watson, 2006 [31], corresponding with Figs 49, 50, and 52 in that reference (-7.3, -7.6, and -8.0 mm from the bregma, respectively). Each effective site is labeled with its experiment number in Panels A-C (when two effective sites were found in a single rat, they are labeled “a” and “b”). Red circles in Panels A-1, B-1, and C-1 correspond with the above images with 2x magnification. Black circles represent sites where DLH was microinjected but did not evoke a change in RSNA (i.e., ineffective sites). Ineffective sites that overlapped locations or were found in sections of the PPT without any effective sites are not shown in Fig 6. Panels D and D-1 shows NADPH-diaphoresis-positive neurons (labeled with a dark blue reaction product) and the Oil Red-O dye used to label the injection track in a section -8.0 mm from the bregma; the effective site is at the most ventral portion of the track. Abbreviations: PnO: nucleus reticularis pontis oralis; SCP: superior cerebellar peduncle; PPT: pedunculopontine tegmentum.

**Fig 7:**
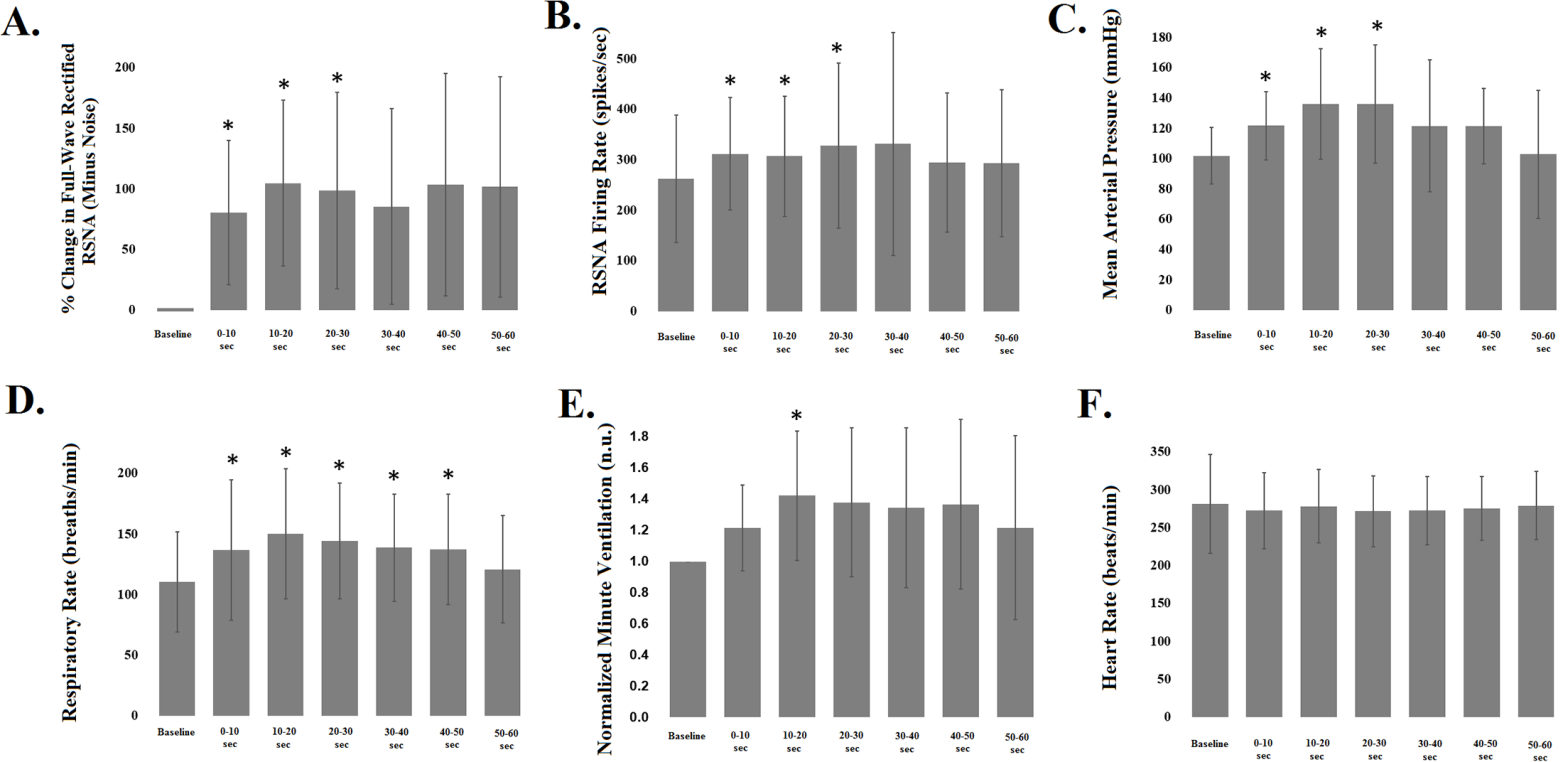
DLH microinjections evoked renal sympathoexcitation, increased mean arterial pressure, and increased respiratory rate. Values are mean ± standard deviation. * denotes values significantly different from baseline. Compared with baseline values, DLH microinjections caused a significant increase in RSNA (percent change from baseline, Panel A; firing rate, Panel B) lasting 30 seconds. Mean arterial pressure increased and remained elevated for 30 seconds (Panel C). Respiratory rate increased significantly, lasting 50 seconds after PPT activation (Panel D). Respiratory minute ventilation was assessed by calculating a normalized tidal volume for each 10-second segment (relative to the baseline period, which was set to 1 n.u.) and multiplying by the respiratory rate. Normalized tidal volume was not statistically different from baseline for any of the 10-second periods (data not shown), but normalized minute ventilation was significantly elevated 10–20 seconds after PPT activation (Panel E). Heart rate did not change significantly (Panel F).

Of a total of 239 pressure microinjections in 18 rats (on average 13.3 DLH microinjections were introduced per rat), 20 renal sympathoexcitatory effective sites were identified. The diagrammatic representations in Fig 6 illustrate the locations of the effective sites throughout the rostral-caudal extent of the PPT, with most identified in dorsal regions of the nucleus. DLH microinjections evoked a significant increase in RSNA, both in the percent change in voltage from baseline and in the firing rate (Fig 7, Panels A and B), which lasted approximately 30 seconds. Responses were accompanied by an increase in mean arterial pressure (to 135.9 ± 36.4 mmHg from 101.6 ± 18.6 [baseline]; Fig 7, Panel C) and an increase in respiratory rate (to 150.0 ± 53.6 breaths per minute from 110.3 ± 41.2 [baseline]; Fig 7 panel D) at 20–30 seconds after PPT activation. Normalized minute ventilation was significantly increased 10–20 seconds after activating the effective site (1.4 ± 0.4 normalized units [n.u., baselines set to 1.0 ± 0]), indicating that both the rate and depth of breathing were briefly altered after microinjecting DLH into effective PPT sites (Fig 7, Panel E). Heart rate was not significantly altered by PPT activation (Fig 7, Panel F).

Many sites (179 DLH microinjections) were encountered in the PPT that did not evoke changes in RSNA, BP, or breathing; the locations of these ineffective areas are marked with open circles in Fig 6 (overlapping circles are omitted to improve readability). Although probing continued in each experiment as long as the rat remained hemodynamically stable, only two experiments identified more than one effective site in the PPT.

## Discussion

The present study demonstrated for the first time that pharmacologic activation of the PPT can evoke renal sympathoexcitation in Nembutal-anesthetized rats. Of the sites tested, 11% evoked increases in RSNA, suggesting that this function may be restricted to a distinct subgroup of PPT neurons. Importantly, PPT activation had an excitatory effect on both RSNA and respiratory parameters. RSNA was significantly elevated for 30 seconds after PPT stimulation, and respiratory rate remained increased for an additional 20 seconds. Two explanations for these observations can be considered: (1) PPT activation evoked hyperventilation, which resulted in reflex activation of RSNA, or (2) the PPT neurons with excitatory projections that activate the renal sympathetic nerves also regulate respiration. If PPT activation increased ventilation and intrathoracic pressure, this could have reflexive consequences for increasing sympathetic activity [36,37]. However, the observations from the present study provide support for the latter argument because PPT activation caused simultaneous increases in RSNA and respiratory rate, while the timing of the pressor effect paralleled the elevation in RSNA. Therefore, we conclude that the change in sympathetic drive caused the BP elevation and that increased RSNA was the direct effect of PPT neuron activation. Interestingly, heart rate remained stable after PPT activation and was not reflexively altered by the longer-lasting changes in ventilation, which may indicate that PPT neurons differentially influence sympathetic outflow.

Data from previous studies demonstrated that pharmacologic activation of the PPT can modulate respiration, but these investigations did not monitor RSNA [15,16,38,39]. The location of PPT activation has been associated with distinct respiratory responses in anesthetized animals [15,16], and respiratory-modulating neurons have been found throughout the PPT. Lydic and Baghdoyan demonstrated respiratory depression in response to electrical stimulation of NADPH-d-positive PPT neurons in Nembutal-anesthetized cats [39]. In a study by Saponjic et al., microinjections of glutamate into the PPT caused anesthetized rats to alternate between tachypnea and bradypnea/apnea [16]. In the present study, however, activation of each sympathoexcitatory effective site was consistently associated with increases in respiratory frequency rather than the decreases or complex/alternating patterns found in other studies [15,16]. Findings from Topchiy et al. support a topographic organization for respiratory-modulating neurons in the PPT; microinjections of glutamate caused apnea when injected near the lateral margins of the PPT, although tachypnea was evoked with injections at the ventrolateral PPT [15].

The neurotransmitter systems involved in the central regulation of sympathetic activities have not been fully defined. Acetylcholine plays a critical role in the regulation of respiration in the PPT and other brainstem nuclei [39]. Because cholinergic neurotransmission is important in BP-regulating connections between the PPT and RVLM [17,18,40,41], and because the RSNA-modulating neurons in the present study were found in areas containing NADPH-d-positive neurons, it is possible that cholinergic PPT neurons can modulate RSNA. Cholinergic PPT neurons already have a well-established role in controlling behaviors that correlate with diverse changes in sympathetic activation, such as REM sleep and arousal [42–45]. Considering that the PPT is a heterogeneous nucleus, the potential contributions of other phenotypes of PPT neurons, including serotonergic and glutamatergic PPT neurons, cannot be ruled out until conducting further investigation.

Studying the PPT in disease neurobiology is a priority area of research considering that deep brain stimulation of the PPT has been used as a treatment for the motor symptoms of Parkinson’s disease [46]. Continued research on the role of PPT neurons in the neural control of kidney function will be important for determining whether deep brain stimulation could evoke unintended renal and cardiovascular responses and to determine how such effects could be optimally managed. Considering that anesthesia can modify sympathetic and cardiovascular responses, barbiturate anesthesia represents an important limitation of the present study [47]. Consistent with the mean heart rate values in the present study, other investigators observed bradycardia in rats receiving barbiturates [48]. Studies in conscious rats will be critical for future investigations of the PPT; chronic models where responses can be measured during wakefulness and during sleep will be particularly informative for determining whether the PPT could represent a therapeutic target for conditions involving sympathetic over-activity and deteriorating renal function. An understanding of how pontine mechanisms contribute to neural control of the kidney is important for treating a variety of human diseases.

## Abbreviations

AUC: Area under the curve
BP: Blood pressure
DLH: DL-homocysteic acid
n.u.: Normalized units
PBS: Phosphate buffered saline
PnO: Nucleus reticularis pontis oralis
PPT: Pedunculopontine tegmentum
REM: Rapid eye movement
RSNA: Renal Sympathetic Nerve Activity
RVLM: Rostral ventrolateral medulla
SCP: Superior cerebellar peduncle

## References

[1] Kannan A, Medina RI, Nagajothi N, Balamuthusamy S. Renal sympathetic nervous system and the effects of denervation on renal arteries. *World J Cardiol*. 2014;6(8):814–823. doi: 10.4330/wjc.v6.i8.814 25228960 10.4330/wjc.v6.i8.814 PMC4163710

[2] O'Donnell CP, Schwartz AR, Smith PL, Robotham JL, Fitzgerald RS, Shirahata M. Reflex stimulation of renal sympathetic nerve activity and blood pressure in response to apnea. *Am J Respir Crit Care Med*. 1996;154(6):1763–1770. 8970368 10.1164/ajrccm.154.6.8970368

[3] Kishi T. Heart failure as an autonomic nervous system dysfunction. *J Cardiol*. 2012;59(2):117–122. doi: 10.1016/j.jjcc.2011.12.006 22341431 10.1016/j.jjcc.2011.12.006

[4] Campos RR, Oliveira-Sales EB, Nishi EE, Paton JF, Bergamaschi CT. Mechanisms of renal sympathetic activation in renovascular hypertension. *Exp Physiol*. 2015;100(5):496–501. doi: 10.1113/expphysiol.2014.079855 25639235 10.1113/expphysiol.2014.079855

[5] Krum H, Schlaich MP, Sobotka PA, Böhm M, Mahfoud F, Rocha-Singh K, et al Percutaneous renal denervation in patients with treatment-resistant hypertension: Final 3-year report of the symplicity HTN-1 study. *Lancet*. 2014;383(9917):622–629. doi: 10.1016/S0140-6736(13)62192-3 24210779 10.1016/S0140-6736(13)62192-3

[6] Adeseun GA, Rosas SE. The impact of obstructive sleep apnea on chronic kidney disease. *Curr Hypertens Rep*. 2010;12(5):378–383. doi: 10.1007/s11906-010-0135-1 20676805 10.1007/s11906-010-0135-1 PMC2975904

[7] Markou N, Kanakaki M, Myrianthefs P, Hadjiyanakos D, Vlassopoulos D, Damianos A, et al Sleep-disordered breathing in nondialyzed patients with chronic renal failure. *Lung*. 2006;184(1):43–49. doi: 10.1007/s00408-005-2563-2 16598651 10.1007/s00408-005-2563-2

[8] Mavanur M, Sanders M, Unruh M. Sleep disordered breathing in patients with chronic kidney disease. *Indian J Med Res*. 2010;131:277–284. 20308753

[9] Grofova I, Keane S. Descending brainstem projections of the pedunculopontine tegmental nucleus in the rat. *Anat Embryol (Berl)*. 1991;184(3):275–290. 1724358 10.1007/BF01673262

[10] Mena-Segovia J, Micklem BR, Nair-Roberts RG, Ungless MA, Bolam JP. GABAergic neuron distribution in the pedunculopontine nucleus defines functional subterritories. *J Comp Neurol*. 2009;515(4):397–408. doi: 10.1002/cne.22065 19459217 10.1002/cne.22065

[11] Datta S, Spoley EE, Patterson EH. Microinjection of glutamate into the pedunculopontine tegmentum induces REM sleep and wakefulness in the rat. *Am J Physiol Regul Integr Comp Physiol*. 2001;280(3):R752–9. 11171654 10.1152/ajpregu.2001.280.3.R752

[12] Rye DB. Contributions of the pedunculopontine region to normal and altered REM sleep. *Sleep*. 1997;20(9):757–788. 9406329 10.1093/sleep/20.9.757

[13] Pienaar IS, van de Berg W. A non-cholinergic neuronal loss in the pedunculopontine nucleus of toxin-evoked parkinsonian rats. *Exp Neurol*. 2013;248:213–223. doi: 10.1016/j.expneurol.2013.06.008 23769975 10.1016/j.expneurol.2013.06.008

[14] Torterolo P, Morales FR, Chase MH. GABAergic mechanisms in the pedunculopontine tegmental nucleus of the cat promote active (REM) sleep. *Brain Res*. 2002;944(1–2):1–9. 12106660 10.1016/s0006-8993(02)02475-7

[15] Topchiy I, Waxman J, Radulovacki M, Carley DW. Functional topography of respiratory, cardiovascular and pontine-wave responses to glutamate microstimulation of the pedunculopontine tegmentum of the rat. *Respir Physiol Neurobiol*. 2010;173(1):64–70. doi: 10.1016/j.resp.2010.06.006 20601208 10.1016/j.resp.2010.06.006 PMC2979313

[16] Saponjic J, Radulovacki M, Carley DW. Injection of glutamate into the pedunculopontine tegmental nuclei of anesthetized rat causes respiratory dysrhythmia and alters EEG and EMG power. *Sleep Breath*. 2005;9(2):82–91. doi: 10.1007/s11325-005-0010-5 15968572 10.1007/s11325-005-0010-5

[17] Padley JR, Kumar NN, Li Q, Nguyen TB, Pilowsky PM, Goodchild AK. Central command regulation of circulatory function mediated by descending pontine cholinergic inputs to sympathoexcitatory rostral ventrolateral medulla neurons. *Circ Res*. 2007;100(2):284–291. doi: 10.1161/01.RES.0000257370.63694.73 17204655 10.1161/01.RES.0000257370.63694.73

[18] Yasui Y, Cechetto DF, Saper CB. Evidence for a cholinergic projection from the pedunculopontine tegmental nucleus to the rostral ventrolateral medulla in the rat. *Brain Res*. 1990;517(1–2):19–24. 2375988 10.1016/0006-8993(90)91002-x

[19] Andresen MC, Doyle MW, Jin YH, Bailey TW. Cellular mechanisms of baroreceptor integration at the nucleus tractus solitarius. *Ann N Y Acad Sci*. 2001;940:132–141. 11458672 10.1111/j.1749-6632.2001.tb03672.x

[20] Cano G, Card JP, Sved AF. Dual viral transneuronal tracing of central autonomic circuits involved in the innervation of the two kidneys in rat. *J Comp Neurol*. 2004;471(4):462–481. doi: 10.1002/cne.20040 15022264 10.1002/cne.20040

[21] Ye D, Guo Q, Feng J, Liu C, Yang H, Gao F, et al Laterodorsal tegmentum and pedunculopontine tegmental nucleus circuits regulate renal functions: Neuroanatomical evidence in mice models. *J Huazhong Univ Sci Technolog Med Sci*. 2012;32(2):216–220. doi: 10.1007/s11596-012-0038-2 22528223 10.1007/s11596-012-0038-2

[22] Institute for Laboratory Animal Research (ILAR). Guide for the care and use of laboratory animals. Washington, D.C. National Academy Press, 2011.

[23] DiBona GF, Jones SY. Analysis of renal sympathetic nerve responses to stress. *Hypertension*. 1995;25(4 Pt 1):531–538. 7721394 10.1161/01.hyp.25.4.531

[24] DiBona GF, Sawin LL. Renal hemodynamic effects of activation of specific renal sympathetic nerve fiber groups. *Am J Physiol*. 1999;276(2 Pt 2):R539–49. 9950935 10.1152/ajpregu.1999.276.2.R539

[25] Jacky JP. Barometric measurement of tidal volume: Effects of pattern and nasal temperature. *J Appl Physiol Respir Environ Exerc Physiol*. 1980;49(2):319–325. 6772618 10.1152/jappl.1980.49.2.319

[26] Seagard JL, Dean C, Patel S, Rademacher DJ, Hopp FA, Schmeling WT, et al Anandamide content and interaction of endocannabinoid/GABA modulatory effects in the NTS on baroreflex-evoked sympathoinhibition. *Am J Physiol Heart Circ Physiol*. 2004;286(3):H992–1000. 14615281 10.1152/ajpheart.00870.2003

[27] Brozoski DT, Dean C, Hopp FA, Hillard CJ, Seagard JL. Differential endocannabinoid regulation of baroreflex-evoked sympathoinhibition in normotensive versus hypertensive rats. *Auton Neurosci*. 2009;150(1–2):82–93. doi: 10.1016/j.autneu.2009.05.243 19464961 10.1016/j.autneu.2009.05.243 PMC3815570

[28] Dean C. Endocannabinoid modulation of sympathetic and cardiovascular responses to acute stress in the periaqueductal gray of the rat. *Am J Physiol Regul Integr Comp Physiol*. 2011;300(3):R771–9. doi: 10.1152/ajpregu.00391.2010 21228344 10.1152/ajpregu.00391.2010

[29] Cleary DR, Phillips RS, Wallisch M, Heinricher MM. A novel, non-invasive method of respiratory monitoring for use with stereotactic procedures. *J Neurosci Methods*. 2012;209(2):337–343. doi: 10.1016/j.jneumeth.2012.06.029 22771713 10.1016/j.jneumeth.2012.06.029 PMC3429636

[30] Brozoski DT, Dean C, Hopp FA, Seagard JL. Uptake blockade of endocannabinoids in the NTS modulates baroreflex-evoked sympathoinhibition. *Brain Res*. 2005;1059(2):197–202. 16154548 10.1016/j.brainres.2005.08.030

[31] Malpas SC. The rhythmicity of sympathetic nerve activity. *Prog Neurobiol*. 1998;56(1):65–96. 9723131 10.1016/s0301-0082(98)00030-6

[32] Paxinos G, Watson C. The rat brain in stereotaxic coordinates. Academic Press, Sydney/New York.

[33] Fink AM, Topchiy I, Ragozzino M, Amodeo DA, Waxman JA, Radulovacki MG, et al Brown norway and zucker lean rats demonstrate circadian variation in ventilation and sleep apnea. *Sleep*. 2014;37(4):715–721. doi: 10.5665/sleep.3576 24899760 10.5665/sleep.3576 PMC4044754

[34] Scherer-Singler U, Vincent SR, Kimura H, McGeer EG. Demonstration of a unique population of neurons with NADPH-diaphorase histochemistry. *J Neurosci Methods*. 1983;9(3):229–234. 6363828 10.1016/0165-0270(83)90085-7

[35] Vincent SR. The ascending reticular activating system—from aminergic neurons to nitric oxide. *J Chem Neuroanat*. 2000;18(1–2):23–30. 10708916 10.1016/s0891-0618(99)00048-4

[36] Dick TE, Hsieh YH, Morrison S, Coles SK, Prabhakar N. Entrainment pattern between sympathetic and phrenic nerve activities in the Sprague-Dawley rat: Hypoxia-evoked sympathetic activity during expiration. *Am J Physiol Regul Integr Comp Physiol*. 2004;286(6):R1121–8. 15001434 10.1152/ajpregu.00485.2003

[37] Sellden H, Delle M, Sjovall H, Ricksten SE. Reflex changes in sympathetic nerve activity during mechanical ventilation with PEEP in sino-aortic denervated rats. *Acta Physiol Scand*. 1987;130(1):15–24. 3296660 10.1111/j.1748-1716.1987.tb08106.x

[38] Saponjic J, Radulovacki M, Carley DW. Respiratory pattern modulation by the pedunculopontine tegmental nucleus. *Respir Physiol Neurobiol*. 2003;138(2–3):223–237. 14609512 10.1016/j.resp.2003.08.002

[39] Lydic R, Baghdoyan HA. Pedunculopontine stimulation alters respiration and increases ACh release in the pontine reticular formation. *Am J Physiol*. 1993;264(3 Pt 2):R544–54. 8457006 10.1152/ajpregu.1993.264.3.R544

[40] Zhang C, Luo W, Zhou P, Sun T. Microinjection of acetylcholine into cerebellar fastigial nucleus induces blood depressor response in anesthetized rats. *Neurosci Lett*. 2016;629:79–84. doi: 10.1016/j.neulet.2016.06.063 27373533 10.1016/j.neulet.2016.06.063

[41] Kumar NN, Ferguson J, Padley JR, Pilowsky PM, Goodchild AK. Differential muscarinic receptor gene expression levels in the ventral medulla of Spontaneously Hypertensive and Wistar-Kyoto rats: Role in sympathetic baroreflex function. *J Hypertens*. 2009;27(5):1001–1008. 19402224 10.1097/hjh.0b013e3283282e5c

[42] Gillin JC, Salin-Pascual R, Velazquez-Moctezuma J, Shiromani P, Zoltoski R. Cholinergic receptor subtypes and REM sleep in animals and normal controls. *Prog Brain Res*. 1993;98:379–387. 8248526 10.1016/s0079-6123(08)62422-x

[43] Van Dort CJ, Zachs DP, Kenny JD, Zheng S, Goldblum RR, Gelwan NA, et al Optogenetic activation of cholinergic neurons in the PPT or LDT induces REM sleep. *Proc Natl Acad Sci U S A*. 2015;112(2):584–589. doi: 10.1073/pnas.1423136112 25548191 10.1073/pnas.1423136112 PMC4299243

[44] Kubin L. Carbachol models of REM sleep: Recent developments and new directions. *Arch Ital Biol*. 2001;139(1–2):147–168. 11256182

[45] Yoshimoto M, Yoshida I, Miki K. Functional role of diverse changes in sympathetic nerve activity in regulating arterial pressure during REM sleep. *Sleep*. 2011;34(8):1093–1101. doi: 10.5665/SLEEP.1168 21804671 10.5665/SLEEP.1168 PMC3138164

[46] Yousif N, Bhatt H, Bain PG, Nandi D, Seemungal BM. The effect of pedunculopontine nucleus deep brain stimulation on postural sway and vestibular perception. *Eur J Neurol*. 2016;23(3):668–670. doi: 10.1111/ene.12947 26800658 10.1111/ene.12947 PMC4819708

[47] Machado BH, Bonagamba LG. Microinjection of L-glutamate into the nucleus tractus solitarii increases arterial pressure in conscious rats. *Brain Res*. 1992;576(1):131–138. 1355385 10.1016/0006-8993(92)90618-j

[48] Murakami M, Niwa H, Kushikata T, Watanabe H, Hirota K, Ono K, et al Inhalation anesthesia is preferable for recording rat cardiac function using an electrocardiogram. Biol Pharm Bull. 2014;37(5):834–9. 24790005 10.1248/bpb.b14-00012

